# Complexity and entropy of natural patterns

**DOI:** 10.1093/pnasnexus/pgae417

**Published:** 2024-09-19

**Authors:** Haoyu Wang, Changqing Song, Peichao Gao

**Affiliations:** State Key Laboratory of Earth Surface Processes and Resource Ecology, Beijing Normal University, Beijing 100875, China; Center for Geographic Analysis, Harvard University, Cambridge, MA 02138, USA; State Key Laboratory of Earth Surface Processes and Resource Ecology, Beijing Normal University, Beijing 100875, China; State Key Laboratory of Earth Surface Processes and Resource Ecology, Beijing Normal University, Beijing 100875, China

**Keywords:** complexity, entropy, natural patterns, complex system

## Abstract

Complexity and entropy play crucial roles in understanding dynamic systems across various disciplines. Many intuitively perceive them as distinct measures and assume that they have a concave-down relationship. In everyday life, there is a common consensus that while entropy never decreases, complexity does decrease after an initial increase during the process of blending coffee and milk. However, this consensus is primarily conceptual and lacks empirical evidence. Here, we provide comprehensive evidence that challenges this prevailing consensus. We demonstrate that this consensus is, in fact, an illusion resulting from the choice of system characterization (dimension) and the unit of observation (resolution). By employing a complexity measure designed for natural patterns, we find that the complexity of a coffee-milk system never decreases if the system is appropriately characterized in terms of dimension and resolution. Also, this complexity aligns experimentally and theoretically with entropy, suggesting that it does not represent a measure of so-called effective complexity. These findings rectify the prevailing conceptual consensus and reshape our understanding of the relationship between complexity and entropy. It is therefore crucial to exercise caution and pay close attention to accurately and precisely characterize dynamic systems before delving into their underlying mechanisms, despite the maturity of characterization research in various fields dealing with natural patterns such as geography and ecology. The characterization/observation (dimension and resolution) of a system fundamentally determines the assessment of complexity and entropy using existing measures and our understanding.

**Significance Statement**

Dynamic systems are ubiquitous in our world, yet our comprehension of their fundamental mechanisms, such as complexity and entropy, remains incomplete and occasionally subjective. Our study uncovers the statistical consistency between complexity and entropy, shedding light on the nature of complexity as a thermodynamically coherent measure of a system. It timely rectifies the prevailing conceptual consensus that complexity initially increases and subsequently decreases during the evolution of a system, as observed in the case of coffee and milk. The way that we observe or characterize a system fundamentally influences the quantification of its complexity and entropy, as determined by our measurements, and ultimately impacts our understanding of the system. We need to reevaluate our approaches to observing the physical world.

## Introduction

For a long time, there seems to have been a conceptual consensus among humans on the (effective) complexity and the disorder (or entropy) of a caffè latte or a system in general (1, 2). It is believed that a caffè latte has increased complexity and entropy when coffee and milk are initially mixed. Then, as a consequence of the second law of thermodynamics, the entropy further increases logarithmically toward a maximum and eventually remains relatively stable (3). In contrast, complexity shows a concave-down trend: the initial increase is followed by a decreasing trend toward a simple state when the coffee and milk are thoroughly mixed (4). Systems with similar consensuses include the diffusion of food dye in water (5), the mixing of coffee and cream (6), and the evolution of the entire universe (7).

Both measures involved in these consensuses are fundamentally necessary to understand a system within and across most disciplines, such as biological systems (8), physical systems (9), media ecosystems (10), cultural systems (11), ecosystems (12), and all learning systems (13). Scientists who utilize these measures typically share the objective of identifying fundamental principles and universal laws that govern the behavior and interactions of a system (14). Complexity has been expected to be an essential measure to understand the structure and to predict the dynamical behavior of a complex system, such as the pattern-forming process in nature (15) and the evolution of life on Earth (16, 17). Entropy is a widely recognized measure of the disorder of a system (18), usually used with complexity (e.g. complexity-entropy plane) (19). It is central to thermodynamics (20), linking with the equilibrium and disequilibrium of a system. It can also be information-theoretic, quantifying the information embedded in a system (21). As a result, both measures have enjoyed enormous scientific attention and wide applications (22). In this case, an accurate and deep understanding of these measures and their relationships is critical.

Here, we advance these understandings by questioning the validity of the preceding conceptual consensus on complexity. Our study further investigates the universal consensus, revealing additional insights that enhance our understanding of complexity and entropy using the quantification approach outlined in Bagrov et al. (5). This complexity measure is designed to be more “observer-independent” compared with traditional complexity metrics, which often require subjective judgments about what constitutes important features versus noise. This aspect is crucial because it allows the complexity measure to be applied consistently across different studies and disciplines without needing significant adjustments or subjective interpretations. Moreover, the complexity measure integrates information across multiple scales, capturing the essential characteristics of the pattern at each scale. By aggregating these scales, the measure provides a more comprehensive understanding of the object's complexity. These features of the measure in Bagrov et al. (5) enhance its applicability and effectiveness, making it an ideal tool for exploring the complex interdependencies within systems ranging from the microscopic to the macroscopic, further enriching our understanding and challenging the existing consensus on complexity and entropy relationships.

Our choice of the complexity measure in Bagrov et al. (5) is motivated by its robustness, versatility, and flexibility. Its robustness is due to its ability to integrate information across multiple scales, making it less susceptible to the influence of any single scale or localized data feature. Its versatility arises from the fact that this complexity measure is not tied to any specific disciplinary background, allowing it to be applied broadly across various fields of study. Its flexibility is highlighted by its capability to handle a wide range of data types, including multiattribute data such as color images and high-dimensional data like 3D Ising models.

In fact, the complexity of a caffè latte (or the diffusion of food dye in Bagrov et al. [5]) does not always increase first and then decrease; under certain conditions, it can even increase logarithmically. Our findings contribute additional insights that complement and expand the understanding established in Bagrov et al. (5), potentially reshaping humans' understanding of the relationship between complexity and disorder/entropy. Toward a new understanding, we then quantified the disorder of all systems involved in Bagrov et al. (5) using information entropy (i.e. Shannon entropy) (23) and thermodynamic entropy (i.e. Boltzmann entropy) (24, 25). Through experimental analysis, we discovered a consistency between complexity and (thermodynamic) entropy. Through theoretical analysis, we further found that the consistency is valid only in a statistical sense, providing explanations for cases when complexity is not consistent with entropy. These discoveries imply a clear gap between humans' conceptual understanding of and our existing approaches for quantifying complexity, similar to a recent gap disclosed on entropy that shocked a whole field (26).

Indeed, the definition (as well as the quantification approach) of complexity varies with the system in question (27, 28). We noted, for example, the computational complexity of an algorithm (29), the social complexity of a region (30), the structural complexity of composite biomaterials and biomineralized particles (31), biological complexity (32), and approximate entropy as a measure of time series complexity (33). This variety holds in the case of entropy, such as the transfer entropy between time series (34) and the permutation entropy of a single time series. Here, we focused on the state of the art and recognized definitions of complexity and entropy for natural patterns, limiting the scope of our conclusions within sciences dealing with spatial data.

## Materials and methods

### Data

All data used for reproducing the experiments of (5) were collected from the Data Availability section of that paper. Simulated data for the mixing of 2 ideal gases were generated in this study. To elaborate on the generation, we explain the simulation of the mixing process. We employed a 3D matrix of 64×64×64 voxels to simulate a closed container. Initially, each voxel at the left half of the container was assigned an attribute of the value 1, representing a molecule of 1 type of ideal gas (or coffee). In contrast, every voxel at the right half was assigned an attribute of zero, representing a molecule of the other type of ideal gas (or milk). The mixing process was then simulated iteratively, with 2,000 being the total number of iterations. At each iteration, we exchanged the positions of each voxel and one randomly selected voxel from the focal voxel's 26 first-order neighbors. The resultant 3D matrix was exported after each iteration, forming the sequence of fields mentioned in the results section.

### Complexity measures and their calculation

All complexity measures were proposed by Bagrov et al. (5). These measures can be calculated for both 2-dimensional (2D) patterns and 3D fields by using the code released by Bagrov et al. (5). The general equation for these complexity measures is as follows:


$$C=\overset{N-1}{\underset{k=0}{\sum }}\;C_{k}=\overset{N-1}{\underset{k=0}{\sum }}\;\left|O_{k+1,k}-\frac{1}{2}(O_{k,k}+O_{k+1,k+1})\right|$$

where $O_{k,k-1}=\frac{1}{L_{k-1}J_{k-1}}\sum_{i=1}^{L_{k}}\sum_{j=1}^{J_{k}}\left(\Lambda^{2}\cdot S_{ij}(k)\cdot S_{ij}(k)\right)$ and $S_{ij}(k)=\frac{1}{\Lambda^{2}}\sum_{m=0}^{\Lambda -1}\sum_{l=0}^{\Lambda -1}S_{\Lambda i-1+m,\Lambda j-1+l}(k-1)$. *N* is the theoretical maximum of the total number of renormalization steps. Λ×Λ is the size of the blocks for renormalization. $L_{k}\times J_{k}$ and $S_{ij}(k)$ are the size of the result by and a vector representing the state of site (i,j) after the *k* -th renormalization step, respectively.

For reproduction and comparison purposes, we followed all practices of Bagrov et al. (5) in applying the equation: We set Λ as 2. In calculating $C_{k\geq 0}$ and $C_{k\geq 1}$, we set a maximum for *k*, namely, k≤N−3.

### Entropy measures and their calculations

The equation for quantifying entropy/disorder in thermodynamics was first proposed by Boltzmann as early as the 1870s (24), but the problems in its application to natural patterns have not been addressed until recent years in landscape ecology (25). In dealing with natural patterns represented by categorical types (i.e. landscape mosaics such as land use/cover maps), the Cushman methods are of use and recommended (35). To address natural patterns represented by gradients such as Bagrov et al. (i.e. landscape gradients in particular and digital images in general) (5), the Gao methods (37) are the only valid choice and were adopted in this study.

Boltzmann entropy and complexity are calculated based on renormalization group transformations, but there is a slight difference. In the transformations by Boltzmann entropy, the size of a coarser representation is smaller than that of the original representation. Technically, such a size change is achieved by aggregating every block of pixels into a single pixel. In contrast, the size is constant in the transformations of complexity: The size of each block is not changed, only the values of pixels in the block are. In this study, the transformations of complexity were adopted in calculating Boltzmann entropy to facilitate fair comparisons.

Because the original method for calculating Boltzmann entropy applies only to 2D natural patterns (37), we developed a version of the calculation method for 3D natural patterns (i.e. fields). The new method shares the same core idea and computational logic as the original method. The only difference lies in the dimension of the renormalization group transformations: The transformations of the original method are 2D, aggregating every 2×2 pixels into macro ones; in contrast, the transformations of the new method are 3D, aggregating every 2×2×2 voxels.

### Compression-based image complexity measures: $IC_{RMSE}$ calculation

The complexity measure $IC_{RMSE}$ evaluates the complexity based on the effects of lossy compression on image quality (49). It is defined as follows:


$$IC_{RMSE}(q)=\frac{RMSE(q)}{CR(q)}$$

where *q* is a quality factor in lossy compression schemes such as JPEG, in which higher values indicate lesser compression and better image quality, RMSE(q) is the root mean square error between the original uncompressed image and the lossy compressed version. CR(q) is the compression ratio, which is defined as the size of the uncompressed image over the size of the compressed image.


$$CR=\frac{S(I)}{S(C(I))}$$

where s(I) represents the file size of the original, uncompressed image *I*, and s(C(I)) denotes the file size of the image after compression by the compressor *C*.

To eliminate the influence of color, all images are first converted into grayscale PNG format before calculation. For non-image data, such as Ising and Heisenberg models, the numerical values are first converted to integers ranging from 0 to 255 and then transformed into grayscale PNG images for computation. In this study, all $IC_{RMSE}$ calculations are based on JPEG compression with a quality factor (q) of 75.

## Results

### Consistency exists between complexity and disorder/entropy

**Fig. 1.**
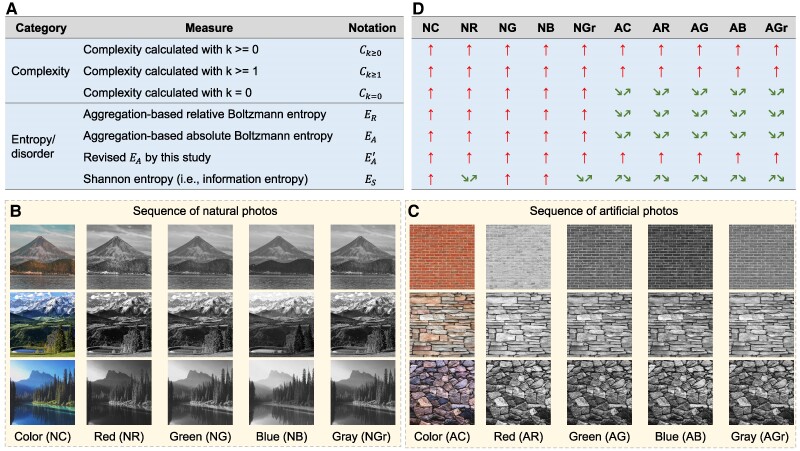
Complexity and entropy of the natural and artificial photos. (A) All measures of complexity and entropy employed in this study. (B) Sequences of natural photos, in which NC, NR, NG, NB, and NGr denote the color, red band, green band, blue band, and grayscale versions of this natural-photo sequence, respectively. (C) Sequences of artificial photos, in which AC, AR, AG, AB, and AGr denote the color, red band, green band, blue band, and grayscale versions of this artificial-photo sequence, respectively. (D) Value trends of each measure for every sequence, in which the symbol ↑ denotes an upward trend. The symbols **↗↘** and **↘↗** denote that the second photo has the greatest and the smallest value among the sequence, respectively. Color (NC) and color (AC) images of 4,096×4,096 pixels were taken from https://www.pexels.com/. Image credits: (A) Pexels/Chriz Luminario, (B) Pexels/kristen munk, (C) Pexels/Daja, (D) Pexels/Pixabay, (E) Pexels/500photos.com, and (F) Pexels/eberhard grossgasteiger.

This discovery was made by reproducing the experiments in the original paper on complexity (5) with entropy measures and by recalculating its complexity measure for comparison. All measures are listed in Fig. 1. In the original paper (5), the complexity measure was calculated with different scale parameters (k≥0, k≥1, and k=0). These parameters were followed in this study, resulting in 3 versions of the complexity measure: $C_{k\geq 0}$, $C_{k\geq 1}$, and $C_{k=0}$ (see Materials and Methods). To quantify disorder, we employed 2 categories of entropy measures. The first category consists of only Shannon entropy ($E_{S}$). In dealing with natural patterns, $E_{S}$ calculated with common methods is widely criticized for its quantification of only the compositional disorder of a system (35). In addition, the resultant Shannon entropy has been demonstrated to be irrelevant to thermodynamics (36). The second category contains the relative and absolute Boltzmann entropies ($E_{R}$ and $E_{A}$) for natural patterns (37), both of which avoid the 2 problems of Shannon entropy. We also slightly revised the renormalization group transformations involved in calculating Boltzmann entropy to make them the same as those with complexity measures (see Materials and Methods), resulting in a revised $E_{A}$ (denoted as $E_{A}^{\prime }$). Before the experiments, we formulated 3 null hypotheses on the consistencies between complexity and entropy measures: $C_{k\geq 0}\propto E_{S}$, $C_{k\geq 1}\propto E_{A}^{\prime }$, and $C_{k=0}\propto E_{R}$.

The first experiment involves 2 sequences of color photos with visually increasing complexity (Fig. 1). One photo sequence is natural patterns and the other is artificial. Because a color photo has 3 bands, its complexity was treated in Bagrov et al. (5) as the sum of each band. This strategy (strategy i) was followed here when calculating Boltzmann entropies. We also introduced 2 more reasonable strategies: we tested the null hypotheses (ii) by using the corresponding grayscale photos and (3) by using every single band of each color photo (Fig. 1). As a result, the first experiment comprised 10 tests as a combination of the following 5 strategies/bands and 2 photo sequences: strategy i (color), ii (gray), strategy iii (red band), strategy iii (green band), and strategy iii (blue band).

All test results can be found in Fig. 1. According to the results with the natural sequence, the values of each complexity/entropy measure exhibit an upward trend in all tests except for that of $E_{S}$ in tests ii and iii (red band). $E_{S}$ first decreases and then increases in these 2 tests, inducing us to reject the null hypothesis of $C_{k\geq 0}\propto E_{S}$. According to the results obtained with the artificial sequence, $C_{k=0}$, $E_{R}$, $E_{A}$, and $E_{S}$ first decrease and then increase. Although such results did not necessitate rejecting any null hypothesis, they demonstrated that $C_{k=0}$ is not a robust measure of complexity.

We further explored the role of the size of the blocks used in the renormalization process. Our expanded tests using different sizes Λ—4, 8, and 16—revealed consistent trends for $C_{k=0}$ across all natural and artificial sequences, thus confirming that in this specific instance, the size of the renormalization blocks does not fundamentally alter the outcome for $C_{k=0}$. However, for a larger Λ value of 64 in artificial sequences, we observed increasing trends in $C_{k=0}$. This variation highlights that while smaller Λ values maintain consistency in complexity measures, larger values can significantly affect the observed trends, primarily due to the lower resolution postrenormalization. From a theoretical perspective, Λ influences the scale difference between the original image and the image after the first renormalization, essentially affecting the resolution. The larger the Λ is the more reduction in resolution there is. Hence, larger Λ values have a more significant impact on the results of complexity. This finding suggests that complexity is influenced by the resolution of observation.

**Fig. 2.**
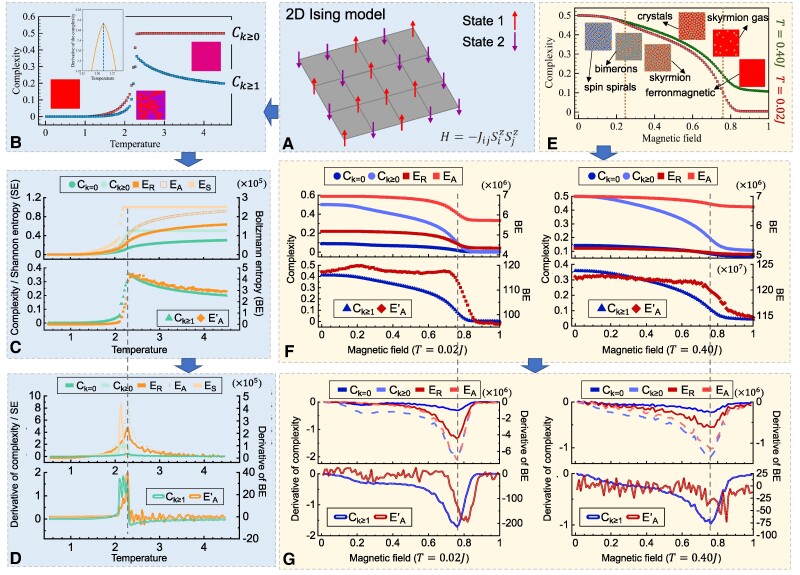
Complexity and entropy of the simulated Ising model and spin textures. (A) Schematic diagram of the 2D Ising model. (B) Complexities ($C_{k\geq 0}$ and $C_{k\geq 1}$) of the simulated 2D Ising model over a temperature range of [0,4.500J] with a step of 0.045J. This figure was adopted from Bagrov et al. (5). (C) The complexity and entropy measures calculated here for each step of the simulated 2D Ising model. (D) Derivatives of these complexity and entropy measures in C. (E) Spin textures simulated in Bagrov et al. (5) by using the Heisenberg model with Dzyaloshinskii–Moriya interactions under temperatures T=0.02J and T=0.40J. This figure was modified from Bagrov et al. (5). (F) The complexity and entropy measures calculated here for the simulated spin textures under temperatures T=0.02J (left) and T=0.40J (right). (G) Derivatives of these complexity and entropy measures in panel F.

The second experiment is the detection of phase transitions in the mathematical model of ferromagnetism in statistical mechanics (i.e. the classical Ising model with nearest-neighbor ferromagnetic interactions on a square lattice of sites and without any external field) (Fig. 2A). Monte Carlo simulations of such a 2D Ising model were performed in Bagrov et al. (5) over a temperature range of [0,4.500J] with a step of 0.045J (Fig. 2B). At each step, a binary image was generated to represent the sites' states. The ($C_{k\geq 1}$)s of these images calculated in Bagrov et al. (5) revealed a critical temperature of 2.26J, which is in agreement with the theoretical analysis (Fig. 2B). Here, we also calculated all complexity and entropy measures of these images (Fig. 2C), revealing consistencies between all pairs (i.e. $C_{k\geq 0}\propto E_{S}$, $C_{k\geq 1}\propto E_{A}^{\prime }$, and $C_{k=0}\propto E_{R}$). Our additional finding is that the detection of the phase transition by $C_{k=0}$ and each Boltzmann entropy ($E_{R}$, $E_{A}$, and $E_{A}^{\prime }$) is more accurate than that by $C_{k\geq 0}$ or $C_{k\geq 1}$. As shown by the derivatives in Fig. 2D, $C_{k=0}$, $E_{R}$, $E_{A}$, and $E_{A}^{\prime }$ have the highest derivatives around the known critical temperature 2.269J. In contrast, $C_{k\geq 0}$ and $C_{k\geq 1}$ only have extreme, rather than the highest, derivatives around the known critical temperature.

The third experiment represents the phase transitions of a more sophisticated nature, or, more precisely, type transitions between spin textures such as spin spirals and skyrmion crystals (38, 39). Such phase transitions were simulated in Bagrov et al. (5) by using the Heisenberg model with Dzyaloshinskii–Moriya interactions (J=1,|D|=1) under a magnetic field (B; Fig. 2E). The simulation was performed for a square lattice of 1,024×1,024 sites under the temperatures T=0.02 and T=0.40J. Under either temperature and by changing B from 0 to 1, one obtains a sequence of spin textures, including spin spirals, bimerons, skyrmion crystals, skyrmion gas, and ferromagnets (Fig. 2E). All these textures were simulated and released by Bagrov et al. (5) as 3-band images, where all pixels have a value range of [−1,1] and a precision of 0.000,001. By calculating all measures and their derivatives of these images (see results from Fig. 2F–G), we found that not only the complexity, but also the entropy measures successfully detected the phase transition from skyrmion crystals to ferromagnets. However, the null hypothesis of $C_{k\geq 0}\propto E_{S}$ should be rejected because $E_{S}$ does not change along with B. No null hypothesis is rejected in this experiment. We note that the Boltzmann entropies of a natural pattern were initially defined for gradients of integers, rather than of decimals, so here we multiplied each pixel value by $10^{6}$ to make it an integer. Such preprocessing does not affect the validity of the consistencies observed in this experiment.

**Fig. 3.**
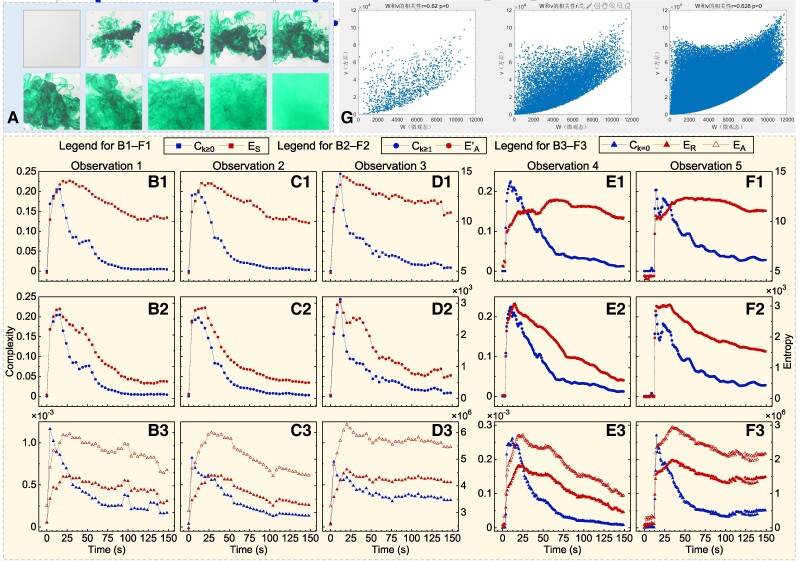
Consistent performance of complexity and entropy measures in the diffusion of food dye and statistical evidence for the consistency. (A) Some of the color photos recorded in Bagrov et al. (5) by observing the diffusion of food dye in water. The diffusion process was independently observed 5 times, recorded as 5 sequences of color photos. Here are only some photos recorded through the first observation. (B–F) Complexity and entropy measures calculated in this study with the color photos recorded through observations 1 to 5. (G) The correlation between the core components of complexity and entropy measures.

The fourth experiment is the diffusion of food dye in water mentioned at the very beginning of this paper; this experiment can be regarded as conceptually equivalent to blending coffee with milk. The process of diffusion was independently observed 5 times by Bagrov et al. (5) and recorded as 5 sequences of color photos (Fig. 3A), each of which has 3 bands of 2,048×2,048 integer pixels with a value range of [0,255]. By calculating the complexity and entropy measures of these 5 sequences, we found that all measures exhibit a concave-down trend (Fig. 3B–F). Based on the results of the first experiment, it is reasonable that the intermediate images in Fig. 3A have higher complexity than those taken at the beginning or end of the diffusion process. We further found that the consistency between $C_{k\geq 1}$ and $E_{A}^{\prime }$ (the average correlation among 5 observations is 0.85) is more profound than that between $C_{k=0}$ and $E_{R}$ (average correlation: 0.80) and that between $C_{k\geq 0}$ and $E_{S}$ (average correlation: 0.59).

As a summary, we have the following findings: Both $C_{k\geq 0}$ and $C_{k\geq 1}$ are suitable to serve as complexity measures, but $C_{k=0}$ is not. This finding confirms the conclusion of Bagrov et al. (5). In addition, $C_{k\geq 0}$ is different from $E_{S}$ because the null hypothesis of $C_{k\geq 0}\propto E_{S}$ should be rejected. However, we experimentally revealed a high consistency between $C_{k\geq 1}$ and the entropy/disorder measure $E_{A}^{\prime }$. More important, this consistency is statistical in essence (proof in Appendix).

### Does entropy truly decrease by blending coffee with milk? No

We can accept the results on complexity in the fourth experiment, as the change in complexity is consistent with our conceptual consensus. However, the evolution of entropy in the experiment is contrary to our conceptual consensus because—according to the second law of thermodynamics—the entropy of the system of coffee and milk should always increase until equilibrium is achieved. Does entropy truly decrease by blending coffee with milk?

Our null hypothesis to the question is no because previous experimental evidence (37, 40) has demonstrated that the entropy indeed increases along with the mixing of 2 ideal gases simulated on a 2D plane. The mixing of 2 ideal gases is conceptually equivalent to that of coffee and milk (by assuming no chemical reactions are involved), but these 2 experiments have a practical difference: The system of coffee and milk was simulated in a 3D space but observed as a 2D plane, resulting in a sequence of images. In this case, the system's entropy should be calculated from a 3D perspective for a field, rather than from a 2D perspective for a plane.

**Fig. 4.**
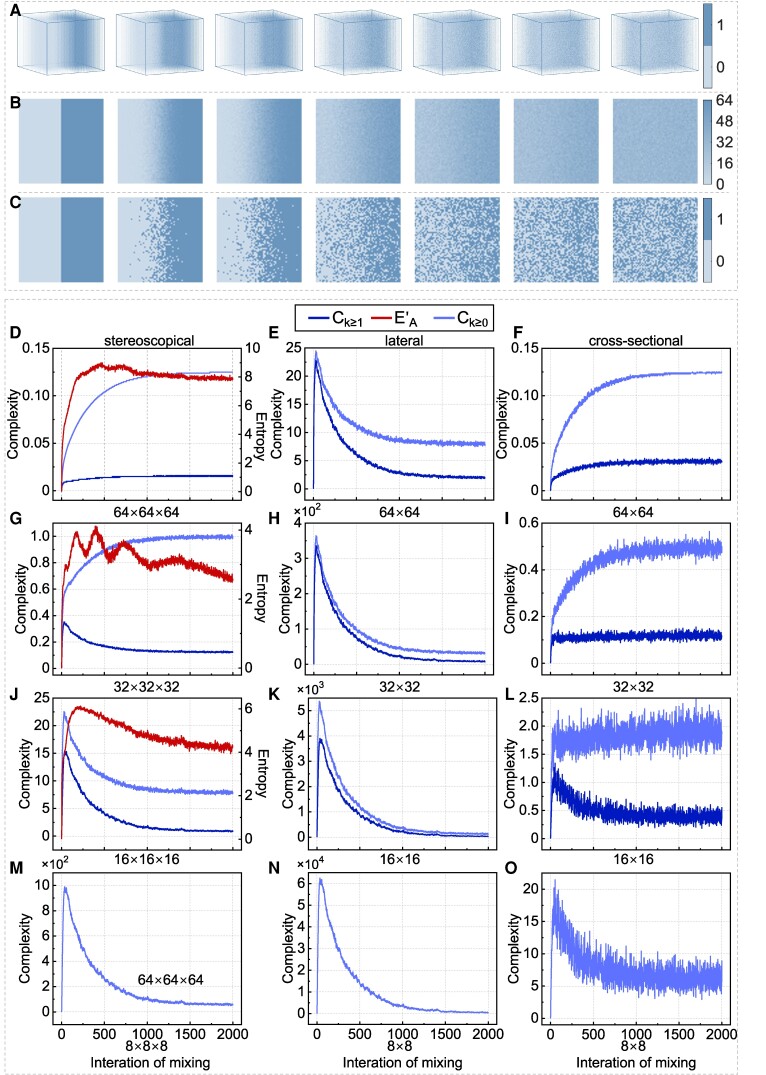
Simulation, observation, complexities, and entropies of the blending of coffee with milk in a container. (A) A part of the simulation results of blending coffee with milk in a 3D container. The simulation was performed iteratively (see Materials and Methods). During each iteration, a field (of voxels) was generated to represent the blending progress. Only some fields are shown here. (B) Lateral observation of the observation process. (C) Cross-sectional observation of the observation process. (D–O) Changes in complexity and entropy with different observations.

To test the null hypothesis, we simulated the blending of coffee with milk in a container and observed the process as a sequence of fields (Fig. 4A; see Materials and Methods). Then, we calculated the Boltzmann entropies of these fields (see Materials and Methods), as shown in Fig. 4D. The entropy indeed increases during the process, which is consistent with the second law of thermodynamics. The results do not induce us to reject our null hypothesis and indicate that entropy does not decrease by blending coffee with milk. The results also demonstrate that the entropy of a system is not equal to that of a 2D picture of the system. Visual observations may mislead us.

### Therefore, why does complexity decrease by blending coffee with milk? An illusion

At this moment, one may wonder why complexity decreases by blending coffee with milk, given that (i) there is a statistical consistency between complexity and entropy and (ii) entropy does not. We find that the decrease in complexity is an illusion to human eyes.

To demonstrate our hypothesis, we calculated the complexity measures of the dynamic, simulated system of coffee and milk, rather than that of the pictures of their blending process. This calculation was achieved by performing 3D renormalization group transformations. In other words, we treated each state of the system as a field, rather than in Bagrov et al. (5) as a color photo of 3 bands; we observed the system stereoscopically, rather than laterally; we calculated the complexity of the system itself, rather than the complexity of what we have seen. Our calculation results, also shown in Fig. 4D, reveal that the complexity never decreases by blending coffee with milk.

Why did the complexity first increase and then decrease in Bagrov et al. (5)? In fact, this decrease is an illusion due to the lateral observation. To simulate this illusion, we first converted the sequence of fields (generated in the previous section) to a sequence of 2D natural patterns by adding up the voxel values at each 2D position along the third dimension (Fig. 4B). As a result, the value range of each pixel of a 2D natural pattern is from 0 to 64. This is also why the snapshots in Fig. 9 of Bagrov et al. (5) have multiple levels of green color. Then, we calculated the complexity measures of this sequence of 2D natural patterns, successfully reproducing the concave-down trend obtained in Bagrov et al. (5). As shown in Fig. 4E, the complexity in this case first increases and peaks when coffee and milk are not thoroughly mixed. After the peak, the complexity exhibits a clear and continuous decreasing trend.

An interesting question, then, is, how does complexity change if we observe the system cross-sectionally? To answer this question, we first extracted the 32nd (randomly selected number) layer along the depth dimension of each field to form a new sequence of 2D natural patterns (Fig. 4C). Each pattern has a size of 64×64 pixels, whose value is either 0 or 1. Then, we calculated the complexity measures of these patterns. The results (Fig. 4F) show that although the complexity exhibits logarithmic growth, the trend is not as smooth as that in the stereoscopic case. The fluctuation is probably because the sum of these values is no longer a constant across the sequence.

In addition to the dimension of observation (e.g. stereoscopically and laterally), we discovered that the resolution of observation also determines the complexity and even its evolution trend. We have observed the simulated system per voxel or pixel, meaning that the resolution of our observation was 64×64×64 or 64×64. As a comparison, we now observe the system with 3 groups of coarser resolutions, i.e. 32×32(×32), 16×16(×16), and 8×8(×8), and then we recalculate the complexity measures. Under a coarser resolution, local patterns within the minimum observation unit (e.g. 2×2 pixels under the resolution of 32×32) are assumed to be homogeneous and reflect the aggregated property (i.e. the sum of local values). According to the results (Fig. 4G–O), we have the following findings:

- The complexity quantified through stereoscopic observation becomes greater but less robust when the observation resolution decreases from 64×64×64 to 32×32×32. If the resolution is further reduced to 16×16×16 or 8×8×8, the evolution trend of complexity even changes from upward to concave-down.
- The complexity quantified through lateral observation shows a concave-down trend across all resolutions.
- The complexity quantified through cross-sectional observation decreases and becomes less robust, along with reduced resolution. Through this observation, complexity shows the most diverse trends of evolution. We obtained upward trends under resolutions of 64×64 and 32×32. When the resolution decreases to 16×16, the complexity increases sharply at the initial stages but fluctuates afterward. However, if the resolution is further reduced to 8×8, the evolution of complexity shows a clear concave-down trend, although with large fluctuations.

In summary, our experiments offer a complementary perspective to the findings of Bagrove et al. (5), confirming that perceived changes in complexity depend significantly on the dimension and resolution of observation. These insights enrich our understanding of complexity in dynamic systems.

### Influence of measurement methods on observed complexity

**Fig. 5.**
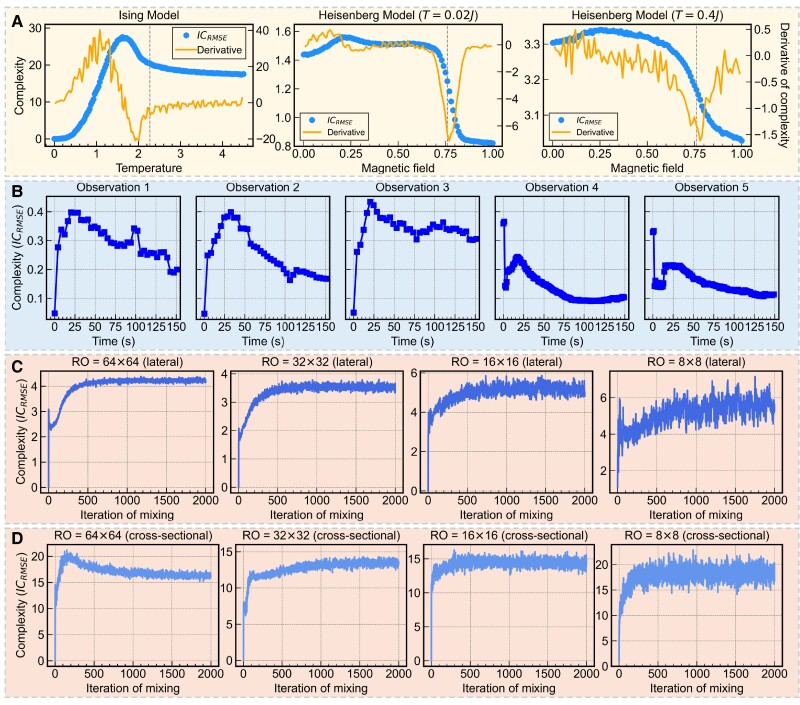
The simulated Ising model and spin textures, the diffusion of food dye, and the results of compression-based complexity ($IC_{rmse}$). (A) $IC_{rmse}$ and its derivatives for the simulated Ising model and Heisenberg model. (B) $IC_{rmse}$ changes for 5 sequences of the diffusion of food dye. (C) $IC_{rmse}$ changes under 64×64, 32×32, 16×16, and 8×8 resolutions for lateral observation. (D) $IC_{rmse}$ changes under 64×64, 32×32, 16×16, and 8×8 resolutions for cross-sectional observation.

We also used a data compression-based complexity measure, $IC_{RMSE}$, to compare results. First, we repeated all previously reported experiments. Because $IC_{RMSE}$ is based on image compression (see Materials and Methods for details), it cannot be directly applied to 3D data; therefore, we only calculated the complexity for 2D data. In the first experiment, for the sequence of natural patterns images, $IC_{RMSE}$ showed an increasing trend. For artificial patterns, however, $IC_{RMSE}$ initially decreased and then increased. This behavior is consistent with the complexity measure $C_{k=0}$, but it differs from the trends observed for $C_{k\geq 1}$ and $C_{k\geq o}$. This suggests that the specific characteristics of the complexity measure can significantly influence the observed trends in complexity. In the second experiment, we examined the 2D Ising model (see Fig. 5A). The $IC_{RMSE}$, for this model exhibited an initial increase followed by stabilization, with a peak around 1.6J. However, it is important to note that the derivative of $IC_{RMSE}$ did not indicate the critical temperature, which is a crucial aspect in detecting phase transitions. This highlights a potential limitation of $IC_{RMSE}$ in capturing certain critical phenomena in complex systems. We extended our analysis to the Heisenberg model in the third experiment, focusing on values of J = 0.02 and J = 0.4. The $IC_{RMSE}$ for the Heisenberg model displayed trends similar to the complexity measure from Bagrov et al. (5). The derivatives of $IC_{RMSE}$ detected phase transitions very close to the actual phase transition points from skyrmion crystals to ferromagnets, though some deviations were observed. This indicates that $IC_{RMSE}$ can capture some aspects of phase transitions.

For the fifth experiment, involving the diffusion of food dye in water, we calculated $IC_{RMSE}$ for all 5 photo sequences (see Fig. 5B). For sequences 1, 2, and 3, $IC_{RMSE}$ initially increased and then decreased, mirroring our previous results for complexity and entropy. However, for sequences 4 and 5, $IC_{RMSE}$ generally followed an initial increase followed by a decrease, except for the photos at the first 2 time points in which the complexity was unexpectedly higher. Upon examining the image file sizes, we found that the file sizes of the photos at the first 2 time points in sequences 4 and 5 (before dye addition) were very small (around 25 KB), whereas the file sizes of the photos at the first 2 time points in sequences 1, 2, and 3 were relatively larger (around 1.7 MB). This difference in initial image file sizes likely influenced the compression results. Consequently, for sequences 4 and 5, the small changes in file size after compression led to higher $IC_{RMSE}$ values, while the larger changes for sequences 1, 2, and 3 resulted in lower $IC_{RMSE}$ values. This finding suggests that $IC_{RMSE}$ is influenced by the properties of the image files themselves, emphasizing the complexity of the file, rather than just the content. It underscores the importance of considering the specific attributes of the data when interpreting complexity measures.

Additionally, we calculated $IC_{RMSE}$ for the simulated coffee and milk blending process. Due to methodological constraints, we focused on lateral and cross-sectional observations, comparing resolutions of 64×64, 32×32, 16×16, and 8×8 (see Fig. 5C and D). The results showed that for both lateral and cross-sectional observations, at any resolution, $IC_{RMSE}$ exhibited an increasing trend throughout the mixing process, without the initial increase followed by a decrease observed in some other measures. This further suggests that the observed trend of increasing then decreasing complexity during the mixing process is a result of specific observation conditions and measurement methods. It highlights the significant impact of system characterization and complexity measurement approaches.

By incorporating these additional experiments and analyses, we aim to provide a more comprehensive understanding of how different complexity measures can influence the interpretation of complexity in various systems. These findings highlight the need for careful consideration of the chosen complexity measure and the specific attributes of the data when drawing conclusions about the behavior of complex systems.

## Discussion

The literature presents diverse understandings of the relationship between complexity and entropy beyond the case of a caffè latte. For example, Meng et al. (41) proposed employing a proxy measure called system sample entropy to quantify the complexity of natural and engineering systems; thus, their underlying hypothesis assumes consistency between entropy and complexity. A similar hypothesis was at the basis of the Coutrot et al. (42) investigation into the effects of environmental complexity on cognitive abilities. In contrast, scientists from crystallography believe that complexity and entropy are mutually reciprocal (43). Therefore, our study's first contribution is discovering the statistical consistency between complexity and entropy, revealing the nature of complexity as a thermodynamically consistent measure of a system.

The second contribution offers a thoughtful adjustment to the widely held belief that complexity increases initially and decreases afterwards along the evolution of a system, typically the system of coffee and milk. Here, we discovered that the complexity does not decrease at all. This discovery adds to the findings of Bagrov et al. (5), which observed a concave-down evolution of complexity in similar experiments. We argue that the observed concave-down evolution is an illusion to human eyes; in essence, it is not about the complexity of the system therein, but rather about the system's characterization. Through experiments, we reproduced the illusion and determined 2 factors of reproduction: the dimension (how we characterize a system) and resolution (how we understand the unit of a system) of an observation.

An important implication of this study is that our observation, or, more precisely, our characterization, of a system fundamentally affects its complexity/entropy quantified by us and eventually its understanding. Although characterization goes beyond a quantification method of the complexity/entropy of natural patterns, it is the first and the easiest step to simplify in many fields when dealing with a real-life system. For example, the first step to understanding landscape dynamics in ecology is to characterize landscapes as landscape patterns. For characterization, the dominant paradigm is a patch-mosaic model [e.g. (44)], which represents landscapes as discrete patches of a categorical variable such as the type of land cover. Such a characterization may lead to a limited understanding of landscape dynamics because it is a lateral observation of landscapes—in terms of this study; this is probably the reason that researchers in ecology are promoting more realistic representations of landscapes by using the gradient model (45).

Another example is geographic information science (46). In this field, geographic space is usually characterized by using a raster or vector model, based on which geographic information systems are established and geographic analysis is performed. In addition to these basic models, advanced models are actively developed for this field to facilitate geo-simulations [e.g. (47)]. This study implies that much attention and caution are still needed in characterizing a system, although characterization research has been considered mature in many fields.

The finding that our experimental results can be affected by observation dimensions reminds us to rethink our observation of this physical world. Our world is multidimensional, but we usually observe only one of its cross-sections, such as the land surface and the stratosphere. In addition, the finding that resolution changes the evolution trend of complexity/entropy emphasizes the determination of the basic units of a system. Although multiple resolutions (or scales) are needed for understanding a system, the resolution showing the basic units of a system could be the most thermodynamically consistent. We consider the world as an example. We need to not only consider it a social-ecological system, but also analyze its social and ecological components.

Notably, the measure of complexity is not and should not be unique, which is also a long consensus: “the definition of complexity cannot be unique” (16). Our measures of complexity and entropy are basic but already state of the art. The complexity measures here are essentially syntactic rather than semantic, although the term “effective complexity” was employed in Bagrov et al. (5) and effective complexity is our long expectation (48).

## Supplementary Material

pgae417_Supplementary_Data

## Data Availability

The data for experiments 1 to 4 have been published by Bagrov et al. (5) and Iakovlev (50). The calculation codes for the complexity measures in Bagrov et al. (5) and Yu and Winkler (49), as well as the entropy in Gao and Li (37), along with the data for the simulated mixing process, are available in https://github.com/CathyW16/MSC_BE/tree/main.
