# Supplementary material for: Complexity and entropy of natural patterns
Source: PNAS Nexus. 2024 Sep 19;3(10):pgae417. doi: 10.1093/pnasnexus/pgae417 (PMC11552627; doi:10.1093/pnasnexus/pgae417)
Supplement: pgae417_Supplementary_Data [file pgae417_supplementary_data.docx]

**
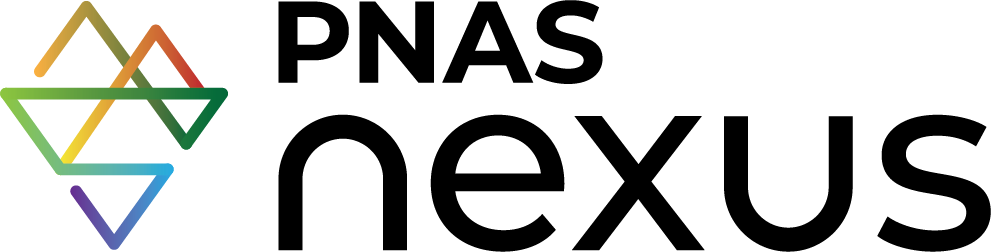
**

**Supporting Information for**

Complexity and entropy of natural patterns

Haoyu Wang^a,b,#^, Changqing Song^a,#^, Peichao Gao^a,^*

^a^State Key Laboratory of Earth Surface Processes and Resource Ecology, Beijing Normal University, Beijing 100875, China; ^b^Center for Geographic Analysis, Harvard University, Cambridge MA 02138, USA

# These authors contributed equally

*Corresponding author: Peichao Gao

**Email:**  gaopc@bnu.edu.cn

**This PDF file includes:**

Supporting text

Supporting Information Text

**Detailed calculations for complexity and entropy measures.** The complexity measure is based on quantifying how much a pattern or structure differs from itself when analyzed at different scales. This is inspired by concepts in theoretical physics, such as renormalization group (RG) theory, which examines how physical systems can be described at various scales. The pattern of interest is subjected to an RG transformation, which involves systematically reducing its scale by coarse-graining. This means that finer details are progressively integrated into a more general view, reducing the resolution of the pattern at each step. At each scale, the transformed pattern is compared with its previous, slightly finer version. The difference between these versions is calculated using a specific mathematical formula:

$$C_{\lambda}=\frac{1}{2}\int_{D} \left( f_{\lambda+d\lambda}\left( x \right)-f_{\lambda}\left( x \right) \right)^{2}dx$$

Here, $f_{\lambda}(x)$ and $f_{\lambda+d\lambda}(x)$ represent the pattern at two successive scales, and $D$ is the domain of the pattern. This formula measures the deviation between the two scales. The deviations calculated for each scale are then summed up to produce the overall complexity measure:

$$C=\sum_{\lambda} C_{\lambda}$$

This summation integrates the complexity contributions from all scales.

For an image of $D\times L$ pixels, there are numerous methods of renormalization. In item (5), the authors chose the simplest discrete decimation scheme. To compute the overlap between two successive scales, $k$ and $k-1$, the coarser pattern (from scale $k$) is rescaled to match the linear size of the finer pattern (from scale $k-1$), ensuring both have the same number of pixels. This rescaling is necessary to compare the patterns accurately.

The overlap between scale $k$ and scale $k-1$, $O_{k,k-1}$​ is given by:

$$O_{k,k-1}=\frac{1}{L_{k-1}J_{k-1}}\sum_{i=1}^{L_{k}} \sum_{j=1}^{J_{k}} \left( \Lambda^{2}\cdot\boldsymbol{S}_{ij}\left( k \right)\cdot\boldsymbol{S}_{ij}\left( k \right) \right)$$

where $\boldsymbol{S}_{ij}\left( k \right)=\frac{1}{\Lambda^{2}}\sum_{m=0}^{\Lambda-1} \sum_{l=0}^{\Lambda-1} \boldsymbol{S}_{\Lambda i-1+m,\Lambda j-1+l}\left( k-1 \right)$. Thus, the complexity is calculated as:

$$C=\sum_{k=Min_{c}}^{Max_{c}} C_{k}=\frac{1}{2}\sum_{k=Min_{c}}^{Max_{c}} \left| f_{k+1}\left( x \right)-f_{k}\left( x \right) \right|^{2}=\frac{1}{2}\sum_{k=Min_{c}}^{Max_{c}} \left| f_{k+1}^{2}\left( x \right)+f_{k}^{2}\left( x \right)-2f_{k+1}\left( x \right) \cdot f_{k}\left( x \right) \right|$$

$$=\sum_{k=Min_{c}}^{Max_{c}} \left| O_{k+1,k}-\frac{1}{2}\left( O_{k,k}+O_{k+1,k+1} \right) \right|$$

where $min_{c}$ and $max\_c$ are the lower and upper bounds of complexity measures for $k$. In this paper, $min_{c}$ is 0 or 1, $max_{c}=N-3$, with $\Lambda=2$. Upon organizing the formulas, the computation of complexity is given by:

$$C=\sum_{k={Min}_{c}}^{{Max}_{c}} C_{k}=\sum_{k={Min}_{c}}^{{Max}_{c}} \sum_{m=1}^{\frac{D}{2^{k+1}}\cdot\frac{L}{2^{k+1}}} \left( \frac{4^{k}c_{k,m}}{D\times L} \right)=\sum_{k={Min}_{c}}^{{Max}_{c}} \sum_{m=1}^{D\times L\div4^{k+1}} \left( \frac{\frac{1}{2}{Var}_{k,m}}{D\times L\div4^{k+1}} \right)$$

where $m$ refers to all the $2\times2$ pixel blocks, and $c_{k,m}$​ represents the difference between the $m$-th block at scale $k$ and the corresponding block at scale $k-1$.

The Boltzmann entropy is calculated using the classic Boltzmann formula, which relates entropy to the logarithm of the number of microstates ($W$):

$$S=k_{B}log(W)$$

Here, $k_{B}$ represents the Boltzmann constant. However, in the context of spatial data, this constant is often set to 1 for simplification, focusing on the logarithmic relationship rather than absolute thermodynamic values. The total number of possible configurations $W$ is computed as the product of possible decompositions for each aggregated macrostate. This captures the combinatorial possibilities across the entire area:

$$W=\prod_{m=1}^{\frac{D}{2}\times\frac{L}{2}} W_{m}$$

where $W_{m}$ here represents the number of possible decompositions of the $m$-th aggregated $2\times2$ pixel blocks, and $\frac{D}{2}\times\frac{L}{2}$ is the total number of aggregated blocks.

Based on the formular of Boltzmann entropy, relative Boltzmann entropy for scale $k$ is:

$$SR(k)=log(\prod_{m=1}^{\frac{D}{2}\times\frac{L}{2}} W_{k,m})$$

The relative Boltzmann entropy ($E_{R}$) used in the paper is that for the first scale:

$$E_{R}=SR(0)$$

Summing up the relative entropies at several levels gives the absolute Boltzmann entropy:

$$E_{A}=\sum_{k={min}_{e}}^{{max}_{e}} SR(k)=\log\left( \prod_{k={min}_{e}}^{{max}_{e}} \prod_{m=1}^{D\times L\div4^{k+1}} W_{k,m} \right)=\sum_{k={Min}_{e}}^{{Max}_{e}} \left( log(\prod_{m=1}^{D\times L\div4^{k+1}} W_{k,m}) \right)$$

The transformations of complexity were adopted in calculating Boltzmann entropy ($E_{A}^{'}$) to facilitate fair comparisons.：

$$E_{A}^{'}=\sum_{k={Min}_{e}}^{{Max}_{e}} \left( \frac{S_{R}\left( L_{k} \right)}{\frac{D}{2^{k+1}}\cdot\frac{L}{2^{k+1}}} \right)=\sum_{k={Min}_{e}}^{{Max}_{e}} \left( \frac{\log\left( {\prod_{m=1}^{\frac{D}{2^{k+1}}\cdot\frac{L}{2^{k+1}}} W_{k,m}} \right)}{D\times L\div4^{k+1}} \right)=\sum_{k={Min}_{e}}^{{Max}_{e}} \sum_{m=1}^{D\times L\div4^{k+1}} \left( \frac{\log\left( W_{k,m} \right)}{D\times L\div4^{k+1}} \right)$$

**Proof of statistical consistency for** $\boldsymbol{C}_{\boldsymbol{k\geq1}}$ **and** $\boldsymbol{E}_{\boldsymbol{A}}^{\boldsymbol{'}}$**.** Although all our experiments revealed the consistency between $C_{k\geq1}$ and $E_{A}^{'}$, we found that this consistency is not always valid, or, more precisely, it is statistical in essence. Moreover, not only $C_{k\geq1}$ but also $C_{k\geq0}$ are statistically positively correlated with $E_{A}^{'}$. Below is our proof.

We first present the mathematical expressions of $C_{k}$ and $E_{A}^{'}$, with the following derivations:

$$C_{k}=\sum_{k={Min}_{c}}^{{Max}_{c}} C\left( L_{k} \right)=\sum_{k={Min}_{c}}^{{Max}_{c}} \sum_{m=1}^{\frac{D}{2^{k+1}}\cdot\frac{L}{2^{k+1}}} \left( \frac{4^{k}c_{k,m}}{D\times L} \right)=\sum_{k={Min}_{c}}^{{Max}_{c}} \sum_{m=1}^{D\times L\div4^{k+1}} \left( \frac{\frac{1}{2}{Var}_{k,m}}{D\times L\div4^{k+1}} \right)$$

$$E_{A}^{'}=\sum_{k={Min}_{e}}^{{Max}_{e}} \left( \frac{S_{R}\left( L_{k} \right)}{\frac{D}{2^{k+1}}\cdot\frac{L}{2^{k+1}}} \right)=\sum_{k={Min}_{e}}^{{Max}_{e}} \left( \frac{\log\left( {\prod_{m=1}^{\frac{D}{2^{k+1}}\cdot\frac{L}{2^{k+1}}} W_{k,m}} \right)}{D\times L\div4^{k+1}} \right)=\sum_{k={Min}_{e}}^{{Max}_{e}} \sum_{m=1}^{D\times L\div4^{k+1}} \left( \frac{\log\left( W_{k,m} \right)}{D\times L\div4^{k+1}} \right)$$

Then, we revealed the statistically positive correlation between the core components of $C_{k}$ and $E_{A}^{'}$, namely, ${Var}_{k,m}$ and $W_{k,m}$ through three random experiments. In the first experiment, we randomly generated 1,000 blocks of $2\times2$ pixels with nonnegative integer values. By calculating all the (${Var}_{k,m}$)s and ($W_{k,m}$)s of these blocks (Fig. 3G), we found that the correlation coefficient between ${Var}_{k,m}$ and $W_{k,m}$ is 0.62 with a $p$-value less than 0.05. In the other two experiments, we increased the number of blocks to 10,000 and 100,000, finding that the correlation became stronger (0.64, $p<0.05$). Therefore, ${Var}_{k,m}$ and $W_{k,m}$ are two statistically positively correlated variables. Because logarithm is a monotonically increasing function, $\frac{1}{2}{Var}_{k,m}$ and $\log\left( W_{k,m} \right)$ are also statistically positively correlated variables.

Based on the preceding correlation, the mathematical proof can be abstracted as the following proposition: We suppose that $E$ and $F$ are two statistically positively correlated variables (correlation coefficient $r>0$). $\left( E_{1}, F_{1} \right)$, $\left( E_{2}, F_{2} \right)$, …, and $\left( E_{h}, F_{h} \right)$ are $h$ independent observations of $\left( E, F \right)$, and their joint probability distribution is identical to that of $\left( E, F \right)$. $E_{e_{1}}$, $E_{e_{2}}$, …, and $E_{e_{p}}$ are $p$ independent observations of $E$. $F_{f_{1}}$, $F_{f_{2}}$, …, and $F_{f_{q}}$ are $q$ independent observations of $F$. Then, $\left( \sum_{i=1}^{h} E_{i}+\sum_{j=1}^{p} E_{e_{j}} \right)=G_{e}$ and $\left( \sum_{i=1}^{h} F_{i}+\sum_{j=1}^{q} F_{f_{j}} \right)=G_{f}$ are positively correlated.

To prove the proposition, we derive the correlation coefficient between $G_{e}$ and $G_{f}$:

$$\rho=\frac{cov \left( G_{e},G_{f} \right)}{\sqrt{Var \left( G_{e} \right)\cdot Var \left( G_{f} \right)}}=\frac{\sum_{i=1}^{h} cov \left( E_{i},F_{j} \right)}{\sqrt{(h+p)\left( h+q \right)\cdot Var\left( E \right)\cdot Var\left( F \right)}}$$

$$=\frac{h\cdot r\cdot\sqrt{Var\left( E \right)\cdot Var\left( F \right)}}{\sqrt{(h+p)\left( h+q \right)}\sqrt{Var\left( E \right)\cdot Var\left( F \right)}}=\frac{h}{\sqrt{(h+p)\left( h+q \right)}}r>0$$

Clearly, $\rho>0$, so the proposition has been proven, as well as the statistical consistency between $C_{k}$ and $E_{A}^{'}$. In addition, the equation of $\rho$ reveals that the consistency is statistically valid even when $C_{k}$ and $E_{A}^{'}$ rely on different renormalization group transformations. The greater the difference in the number of renormalization steps is, the lower the correlation.
